# Criminal charges prior to and after initiation of office-based buprenorphine treatment

**DOI:** 10.1186/1747-597X-7-10

**Published:** 2012-03-19

**Authors:** Elizabeth E Harris, Janet Soeffing Jacapraro, Darius A Rastegar

**Affiliations:** 1 Albert Einstein College of Medicine/Montefiore Medical Center, 1621 Eastchester Road Bronx, New York 10461, USA; 2 Health Care for the Homeless, 421 Fallsway, Baltimore, MD 21202, USA; 3 Johns Hopkins School of Medicine, Division of Chemical Dependence, Johns Hopkins Bayview Medical Center, 5200 Eastern Avenue, Baltimore, MD 21224, USA

**Keywords:** Opioid-related disorders, Crime, Primary health care, Buprenorphine

## Abstract

**Background:**

There is little data on the impact of office-based buprenorphine therapy on criminal activity. The goal of this study was to determine the impact of primary care clinic-based buprenorphine maintenance therapy on rates of criminal charges and the factors associated with criminal charges in the 2 years after initiation of treatment.

**Methods:**

We collected demographic and outcome data on 252 patients who were given at least one prescription for buprenorphine. We searched a public database of criminal charges and recorded criminal charges prior to and after enrollment. We compared the total number of criminal cases and drug cases 2 years before versus 2 years after initiation of treatment.

**Results:**

There was at least one criminal charge made against 38% of the subjects in the 2 years after initiation of treatment; these subjects were more likely to have used heroin, to have injected drugs, to have had any prior criminal charges, and recent criminal charges. There was no significant difference in the number of subjects with any criminal charge or a drug charge before and after initiation of treatment. Likewise, the mean number of all cases and drug cases was not significantly different between the two periods. However, among those who were opioid-negative for 6 or more months in the first year of treatment, there was a significant decline in criminal cases. On multivariable analysis, having recent criminal charges was significantly associated with criminal charges after initiation of treatment (adjusted odds ratio 3.92); subjects who were on opioid maintenance treatment prior to enrollment were significantly less likely to have subsequent criminal charges (adjusted odds ratio 0.52).

**Conclusions:**

Among subjects with prior criminal charges, initiation of office-based buprenorphine treatment did not appear to have a significant impact on subsequent criminal charges.

## Introduction

Substance dependence is a common medical problem and is associated with criminal activity [1,2]. It is estimated that nearly 6 million individuals are arrested each year in the United States and that nearly one half meet criteria for a substance use disorder, but most do not receive treatment [3]. One of the goals of addiction treatment is to reduce criminal activity. A number of studies have reported a decrease in rates of crime when opioid-dependent individuals are enrolled in opioid agonist treatment, both when compared to the period prior to enrollment [4-7], and when compared to those who are not enrolled in opioid agonist treatment [8-10]. However, all of these studies involved subjects who were on methadone maintenance therapy. While office-based buprenorphine treatment has been shown to be an effective treatment for opioid dependence [11], there are no published studies of the effect of office-based buprenorphine maintenance therapy on criminal activity [12].

Given the prevalence of substance use in the criminal justice population [13] and the current limited access to pharmacologic treatment for opioid dependence, there is a tremendous need for effective treatment; incarceration may be an opportunity to initiate treatment for opioid dependence and improve rates of recidivism [14,15]. Buprenorphine maintenance therapy may be more acceptable than methadone to criminal offenders released from prison, because of the greater flexibility of receiving prescribed buprenorphine rather than attending a methadone maintenance program on a daily basis [16]. However, subjects with a criminal history may benefit from the more intensive treatment and monitoring that a methadone program offers. Moreover, a criminal history may be associated with poorer treatment retention [17], although one recent study found that a history of incarceration was not associated with poorer outcomes in office-based buprenorphine treatment [18].

The goal of this study is to determine the impact of buprenorphine maintenance therapy in a primary care setting on rates of criminal charges and to examine risk factors for criminal charges in the 2 years after initiation of treatment.

## Methods

### Setting

The Comprehensive Care Practice is a primary care clinic on the Johns Hopkins Bayview Medical Center campus which is staffed by 5 internists, a nurse practitioner, and 3 internal medicine residents who share a panel of patients. The practitioners provide general primary care, with a focus of serving patients with HIV infection and/or substance use disorders.

Visits for opioid dependence occur as routine primary care visits. A more detailed description of this clinic's treatment practices has been published previously [19]. Briefly, there is no uniform protocol or dosing regimen, with buprenorphine doses ranging from 2 to 32 mg, with most patients on 8-16 mg daily. Induction occurs at home or in the office and follow-up occurs weekly to monthly, based on the provider's discretion; patients are usually seen more frequently early in treatment or when there is continued substance use. Treatment is continued or discontinued based on the provider's discretion. The practice does not provide any additional onsite psychosocial services and patients are referred to community resources.

### Subjects

The study included all patients who had received at least one prescription for sublingual formulation of buprenorphine from August 2003 to September 1, 2007.

### Data collection

As part of a previously performed study [19], a database of all patients who received at least one prescription for buprenorphine during this period of time had already been created. Data were collected retrospectively from the patient medical records. Demographic information recorded included age, gender, type of insurance and employment status. Substance abuse history collected included substances used and history of injection drug use. We also collected data on recent (within 30 days) drug treatment, including methadone and buprenorphine maintenance. Also recorded were relevant comorbidities (hepatitis C, HIV, chronic pain and chronic psychiatric illness).

The time period after receiving the first prescription was divided into twelve monthly blocks for the purposes of outcomes analysis. Patients were considered to be in treatment for each block in which they were prescribed buprenorphine at any point. There was no fixed protocol for collection of urine drug tests, so for each month in which the patient was receiving treatment, patients were classified as "opioid-positive" or "opioid-negative". Patients were classified as "opioid-positive" if any of their urine drug tests during that month were positive for opioids (other than those prescribed), if they reported using other non-prescribed opioids, or if a urine drug test was not collected and their most recent test was positive. Patients were classified as "opioid-negative" if all urine drug tests collected during that month were negative for opioids (other than those prescribed), or if the provider decided not to collect a test and their most recent one was negative.

We utilized the Maryland Judiciary Case Search website http://casesearch.courts.state.md.us/inquiry/inquiry-index.jsp to determine whether subjects had any criminal charges and the number of cases filed against each subject. This database includes data on all criminal charges in the state of Maryland since 1991. The database provides the defendant name, city and state, date of birth, trial date, charges, and case disposition. We searched this database by each patient's name and birthdate, and recorded whether the subject had ever had criminal charges prior to initiating treatment. We tabulated the total number and types of criminal cases two years prior to the date of the first prescription for buprenorphine, and for the two-year period of time after that date. We counted cases as listed separately on the website, not individual charges; we decided to do this because many cases included multiple related charges (for example, multiple charges of drug possession with separate charges for possession of drug paraphernalia). We did not count cases that were limited to motor vehicle charges, with the exception of driving under the influence of alcohol. We classified criminal cases as "drug cases" or "other"; "drug cases" included any cases with charges of possession or distribution of controlled dangerous substances (CDS).

### Statistical analysis

Bivariate analysis was used to compare demographic factors and outcomes among subjects with and without criminal charges in the two years after initiation of treatment. We also used bivariate analysis to compare the mean number of criminal cases in the two years before and after initiation of treatment. We also compared the number of criminal cases before and after initiation of treatment among subjects who remained in treatment at 12 months and those who were opioid negative for 6 or more months. Chi-square tests were used to analyze categorical variables and paired t tests for continuous variables. Wilcoxon signed ranks and Mann Whitney U tests were used to compare variables that were not normally distributed. P values less than 0.05 were considered statistically significant. Multivariate analysis was used to analyze factors associated with criminal charges after initiation of treatment. Variables with a p value < 0.1 were entered into the logistic regression model; for factors that were highly co-linear (Pearson's correlation coefficient > 0.4), only one was entered. Analysis was performed using PASW software (version 18). This study was approved by the Johns Hopkins Institutional Review Board.

## Results

The study included 252 patients who had been given at least one prescription for sublingual formulation of buprenorphine from August 2003 to September 1, 2007. Two subjects from the original cohort were not included because they could not be identified due to gaps in record-keeping.

The number of subjects with at least one criminal charge in the two years after initiating treatment was 97 (38.4%). Table 1 provides demographic and outcome data on subjects with and without criminal charges during this period. A number of characteristics were associated with criminal charges after enrollment in buprenorphine maintenance therapy. Those who reported a history of heroin abuse were more likely to have had criminal charges, while those who reported prescription drug abuse were less likely to have been charged. Hepatitis C virus (HCV) infection and injection drug use (IDU) were also associated with criminal charges. Subjects with criminal charges were less likely to have been receiving opioid maintenance treatment within 30 days prior to initiating treatment at this practice. All of the subjects with criminal charges in the two years after initiating treatment had prior criminal charges, compared to only 67.7% (p < 0.001) of those whom had no criminal charges after initiating treatment; these subjects were also more likely to have had charges in the 2 years prior to initiation of treatment and had a significantly higher median number of prior cases. Subjects with criminal charges in the two years after starting treatment were less likely to achieve ≥ 6 opioid-negative months; they were also less likely to remain in treatment at 12 months, but this difference was not statistically significant.

**Table 1:** Comparison of subjects with and without criminal charges in the 2 years after initiation of office-based buprenorphine treatment

Characteristic	Criminal charges (N = 97)	No criminal charges (N = 155)	Χ^2^, df	P-value^a^
Median Age (range)	40 (20-56)	41 (18-67)		0.770^b^

Sex				
Male	56 (57.7%)	86 (55.5%)	0.12, 1	0.726
Female	41 (42.3%)	69 (44.5%)		

Insurance				
Commercial	38 (39.2%)	66 (42.6%)	0.29, 1	0.593
Medicaid	36 (37.1%)	53 (34.2%)	0.22, 1	0.637
Medicare	19 (19.6%)	23 (14.8%)	0.04, 1	0.839
None	4 (4.1%)	4 (2.6%)	0.46, 1	0.497

Employment Status				
Employed	38 (39.2%)	75 (48.4%)	2.07, 1	0.153
Unemployed	31 (32.0%)	43 (27.7%)	0.51, 1	0.457
Disabled	28 (28.9%)	37 (23.9%)	0.78, 1	0.378

Abused Substances				
Heroin	90 (92.8%)	119 (76.8%)	10.81, 1	**0.001**
Opioid Rx	15 (15.5%)	57 (36.8%)	13.28, 1	**< 0.001**
Cocaine	58 (59.8%)	76 (49.0%)	0.003, 1	0.096
Alcohol	16 (16.5%)	26 (16.8%)	2.78,1	0.954
Benzodiazepines	8 (8.2%)	15 (9.7%)	0.15, 1	0.701

Recent opioid maintenance treatment	18 (18.6%)	50 (32.3%)	5.69, 1	**0.017**

IDU	67 (69.1%)	85 (54.8%)	5.05, 1	**0.025**

Co-morbidities				
HIV	14 (14.4%)	22 (14.2%)	0.003, 1	0.958
HCV	58 (59.8%)	67 (43.2%)	6.55, 1	**0.010**
Psychiatric	102 (51.3%)	23 (43.4%)	1.60, 1	0.309
Chronic Pain	35 (17.6%)	11 (20.8%)	0.82, 1	0.596

Any prior criminal charges	97 (100%)	105 (67.7%)	39.04, 1	**< 0.001**

Median number of prior criminal cases (range)	9 (1-43)	1 (0-25)		**< 0.001**^b^

Any criminal charges in past 2 years	62 (63.9%)	46 (29.7%)	28.56, 1	**< 0.001**

≥ 6 opioid-negative mos.	34 (35.1%)	83 (53.5%)	8.21, 1	**0.004**

12 mos. in treatment	46 (47.4%)	93 (60.0%)	3.82, 1	0.051

IDU: injection drug use, HCV: hepatitis C infection, HIV: human immunodeficiency virus infection, df: degrees of freedom

^a ^Pearson chi-square test unless otherwise indicated; ^b ^Mann Whitney *U *test

As shown in Table 2, there was a small decline in the proportion of subjects with at least one criminal charge in the two years before and after initiation of treatment, but this was not statistically significant. Likewise, there was no significant difference in the mean number of criminal cases in the two years before and after enrollment; before enrollment, there was a mean of 0.77 cases per subject, compared to 0.70 cases after enrollment. Among patients who remained in treatment at 12 months, there was no significant difference in the number of criminal cases before and after enrollment. However, among subjects who had ≥ 6 opioid-negative months, there was a significant decline in the mean number of criminal cases.

**Table 2:** Overall charges and drug charges in the 2 years before and after initiation of buprenorphine treatment

	2 years before treatment initiation	2 years after treatment initiation	P value
Overall charges			

Number of subjects with a charge (percentage)	108 (42.9%)	97 (38.5%)	0.22^a^

Mean number of cases per subject (SD)	0.77 (1.21)	0.70 (1.15)	0.37^b^

Mean number of cases among subjects in treatment for 12 months (SD)	0.71 (1.25)	0.60 (1.08)	0.38^b^

Mean number of cases among subjects opioid negative ≥ 6 months (SD)	0.67 (1.19)	0.43 (0.78)	**0.03**^b^

Drug charges			

Number of subjects with a drug charge (percentage)	54 (21.4%)	65 (25.8%)	0.11^a^

Mean number of drug cases per subject (SD)	0.31 (0.68)	0.35 (0.70)	0.46^b^

Mean number of drug cases among subjects in treatment for 12 months (SD)	0.30 (0.71)	0.34 (0.74)	0.60^b^

Mean number of drug cases among subjects opioid negative ≥ 6 months (SD)	0.28 (0.72)	0.21 (0.48)	0.27^b^

SD-standard deviation

^a ^Pearson chi-square test; ^b ^Wilcoxon signed ranks test

Similar to the overall charges, there was no significant difference in the proportion of subjects with at least one drug charge. There was also no significant difference in the mean number of drug cases before or after enrollment. Likewise, there was no significant difference in the mean number of drug cases among subjects who remained in treatment at 12 months or among those who achieved ≥ 6 opioid-negative months.

On multivariate analysis, charges in the past 2 years was the strongest predictor of charges in the 2 years after initiation of treatment, as shown in Table 3. Subjects who were receiving opioid maintenance treatment within 30 days of initiating treatment at this practice were less likely to have had subsequent charges. None of the other factors was significantly associated with criminal charges in the two years after initiation of treatment.

**Table 3:** Multivariate analysis of factors associated with criminal charges in the 2 years after initiation of office-based buprenorphine treatment

Factor	Adjusted Odds Ratio (95% confidence interval)	P value^a^
Recent charges (past 2 years)	3.893 (2.216-6.839)	**< 0.001**

Recent opioid maintenance	0.515 (0.265-0.998)	**0.049**

≥ 6 months opioid negative	0.597 (0.335-1.063)	0.080

Heroin abuse	2.479 (0.897-6.994)	0.086

Cocaine abuse	1.459 (0.804-2.648)	0.214

Injection drug use	1.186 (0.618-2.275)	0.609

The following factors were not included in the multivariate analysis due to a high correlation with another factor (indicated in parenthesis): any prior charges (recent charges), remaining in treatment at 12 months (≥ 6 months opioid negative), prescription opioid abuse (heroin abuse), hepatitis C (injection drug use).

^a ^P values calculated by Wald chi-square tests in a logistic regression model; degrees of freedom = 1 for each

## Discussion

In this cohort, enrollment in office-based buprenorphine treatment did not seem to result in a significant decline in criminal charges. There likewise was no significant difference in the proportion of subjects who had a drug charge and no significant difference in the mean number of drug cases. However, among the 46% of subjects who were more successful in treatment, defined as ≥ 6 opioid-negative months in the first year after initiation of treatment, there was a significant decline in criminal cases.

This is the first study to our knowledge that looks at the effect of office-based buprenorphine therapy on criminal activity. Initiation of buprenorphine maintenance therapy did not seem to result in a significant decrease in criminal charges, including drug charges. This is in contrast to several studies of subjects in methadone maintenance treatment [4-7]. There are a few possible reasons for these differences. One is that we included all enrollees into our study, not just those who remained in treatment; however, we did not see a significant decline when limiting our analysis to those who remained in treatment at one year. Another difference is that we used an objective measure rather than self-report. Yet another difference is that subjects in this primary care setting received less intensive monitoring than what is typically provided in methadone maintenance programs. It is possible that these subjects would do better in programs that monitor them closely and provide more psychosocial support.

While treatment retention did not affect rates of criminal charges in our cohort, those who had ≥ 6 opioid-negative months did have a significant decline in criminal charges, suggesting that there was a subgroup in which the treatment was associated with changes in criminal behavior. Other studies have shown that larger reductions in crime are associated with abstinence from opioids. For example, a large study done in England, the National Treatment Outcome Research Study (NTORS), used a national database of convictions and found that there was a significant decline in convictions at 5 years, and that this was associated with reductions in heroin use [20].

Our findings may be relevant to the treatment of recently-incarcerated individuals. Previous studies have shown buprenorphine treatment to be effective in this population and it may be better accepted than methadone maintenance [16]. However, if reduction in criminal activity is a goal, then a referral to office-based buprenophine treatment alone may not be sufficient. This needs to be studied further and other strategies developed to address the needs of this population.

One factor that may have attenuated the impact of this treatment was that approximately one-fourth of the subjects had been on opioid maintenance treatment (methadone or buprenorphine) within 30 days of initiating treatment. While those who had recently been on opioid maintenance treatment were less likely to have subsequent criminal charges, when we excluded these subjects from our analysis, we likewise did not find any significant differences in number of criminal cases (total or drug cases) before and after initiation of treatment (data not shown).

A limitation of our study is that we looked only at criminal charges; not all criminal activity leads to criminal charges and not all charges are necessarily indicative of criminal activity (i.e., some may have been wrongly charged). On the other hand, criminal charges are an objective measure and likely correlate with criminal activity. These rates may have been affected by changes in police or criminal justice policies; however, Federal Bureau of Investigation statistics indicate that there has been a decline in crime and arrest rates in Maryland between 2001 and 2009 [21]; therefore, it is unlikely that the absence of a significant decline was due to more aggressive policing or enforcement. Another limitation is that we only looked at criminal charges recorded in Maryland, and could not include crimes committed in other states. However, unlike many other studies, we used an "intention-to-treat" model and included everyone who had received at least one prescription for buprenorphine, not just those who remained in treatment.

In summary, our study suggests that a referral to office-based buprenorphine treatment alone is not sufficient to address criminal behavior among subjects with opioid dependence and a history of criminal charges. It is possible that some of these individuals may benefit from more intensive treatment and monitoring than can be provided in a primary care setting.

## Competing interests

The authors declare that they have no competing interests.

## Authors' contributions

All authors contributed to the design and the manuscript preparation and have approved the final manuscript. Data collection was conducted by JSJ and EEH, analysis was performed by DAR.
